# Role of robotic natural orifice specimen extraction surgery in colorectal neoplasms

**DOI:** 10.1038/s41598-021-89323-z

**Published:** 2021-05-10

**Authors:** Hongliang Yao, Tiegang Li, Weidong Chen, Sanlin Lei, Kuijie Liu, Bo Liu, Jiangjiao Zhou

**Affiliations:** Department of General Surgery, The Second Xiangya Hospital of Central South University, Changsha, 410011 China

**Keywords:** Gastroenterology, Oncology

## Abstract

Natural orifice specimen extraction surgery (NOSES) is especially suitable for colorectal surgery. Until now, most of the reports published were about laparoscopic NOSES, the reports about robotic NOSES are extremely rare. This study aims to explore the safety and feasibility of robotic NOSES for colorectal neoplasms. All patients underwent robotic NOSES from March 2016 to October 2019 in our hospital were enrolled for retrospective analysis. Clinicopathological data including patient characteristics, perioperative information and pathological information were collected and analyzed. According to the distance between tumor and anus or whether neoadjuvant chemoradiotherapy (nRCT) is performed, we grouped the cases and studied its influence on robotic NOSES. Also, we compared the previous reports on laparoscopic NOSES with our study and revealed advantages of robotic NOSES in terms of safety and feasibility. A total of 180 patients were enrolled. The average distance from the lower edge of the tumor to the anus was (8.64 ± 3.64) cm and maximum circumferential diameter (CDmax) of specimen was (3.5 ± 1.6) cm. In terms of safety, the average operation time, intraoperative blood loss, and postoperative hospital stay were (187.5 ± 78.3) min, (47.4 ± 34) mL, and (11.3 ± 7.5) days, respectively. In terms of feasibility, the average number of lymph node harvested was (14.8 ± 5). Robotic NOSES shows advantages in terms of safety and feasibility compared with laparoscopic NOSES. This procedure could not only be a safe procedure but also could achieve good oncological outcomes.

## Introduction

Total mesorectal resection (TME) and laparoscopic surgery are the major breakthroughs for colorectal cancer surgery^1^. However, an incision must still be made at the abdominal wall to remove the specimen^2^. Natural orifice specimen extraction robotic surgery (NOSES) can solve this problem. NOSES is designed to use laparoscopic instruments or soft endoscopy and other equipment to complete intra-abdominal surgery. No auxiliary incision is made at the abdominal wall, and the specimen is removed through the natural lumen (anus or vagina). Compared with traditional laparoscopic surgery, the most evident advantage of NOSES is the avoidance of auxiliary incision, which could meet the esthetic needs of patients, such as actors and gymnasts, engaged in specific occupations. To date, NOSES can be used for colorectal, stomach, small intestine, hepatobiliary, and gynecological tumors^3^. Many reports had also verified the effectiveness and safety of NOSES for colorectal neoplasms^4,5^.

Although the procedure of NOSES could be performed by laparoscopy, the da Vinci Surgical System still has its unique advantages due to its high-definition field of vision and seven degrees of freedom of the operation arm. Our department is one of the centers which previously carried out robotic NOSES for colorectal cancer in China^6^. This study aimed to summarize single-center experience in robotic NOSES for sigmoid and rectal neoplasms through a retrospective analysis of the collected cases.

## Materials and methods

### Inclusion and exclusion criteria of cases

From March 2016 to October 2019, all patients with sigmoid and rectal neoplasm and underwent robotic NOSES in the General Surgery Department of The Second Xiangya Hospital, Central South University were considered for retrospective analysis.

Inclusion criteria: (1) age ≥ 18 years old; (2) diagnosis of sigmoid and rectal neoplasm by biopsy via colonoscopy or benign neoplasm located in rectum that could not be resected locally through the anus; (3) written informed consent of patients; (4) expected removal of the specimen through the anus indicated by preoperative evaluation.

Exclusion criteria: (1) emergency operation due to gastrointestinal obstruction, perforation, or bleeding; (2) metastasis of the lung, bone, or liver that cannot be removed simultaneously; (3) contraindications for robotic surgery.

This work is in accordance with the declaration of Helsinki and is approved by the Ethics Committee of the Second Xiangya Hospital, Central South University.

### Information collection

The statistical information included the following: (1) patient characteristics: gender, age, chief complaint, comorbidity, history of laparotomy, body mass index (BMI), American Society of Anesthesiologists (ASA) classification, distance from the lower edge of the tumor to the anus (lower group < 5 cm, 5 ≤ middle group < 10 cm, upper group ≥ 10 cm)^7^, and presence or absence of nRCT (surgery was performed 4–6 weeks after nRCT); (2) perioperative information: operation time, intraoperative blood loss, protective ileostomy, conversion to laparotomy, postoperative hospital stay, postoperative complications, reoperation, and total costs; (3) pathological information: histological type, differentiation, maximum circumferential diameter (CDmax) of specimen, depth of tumor invasion, the number of lymph nodes harvested, and the number of metastatic lymph nodes.

### Surgical procedure

**Figure 1:**
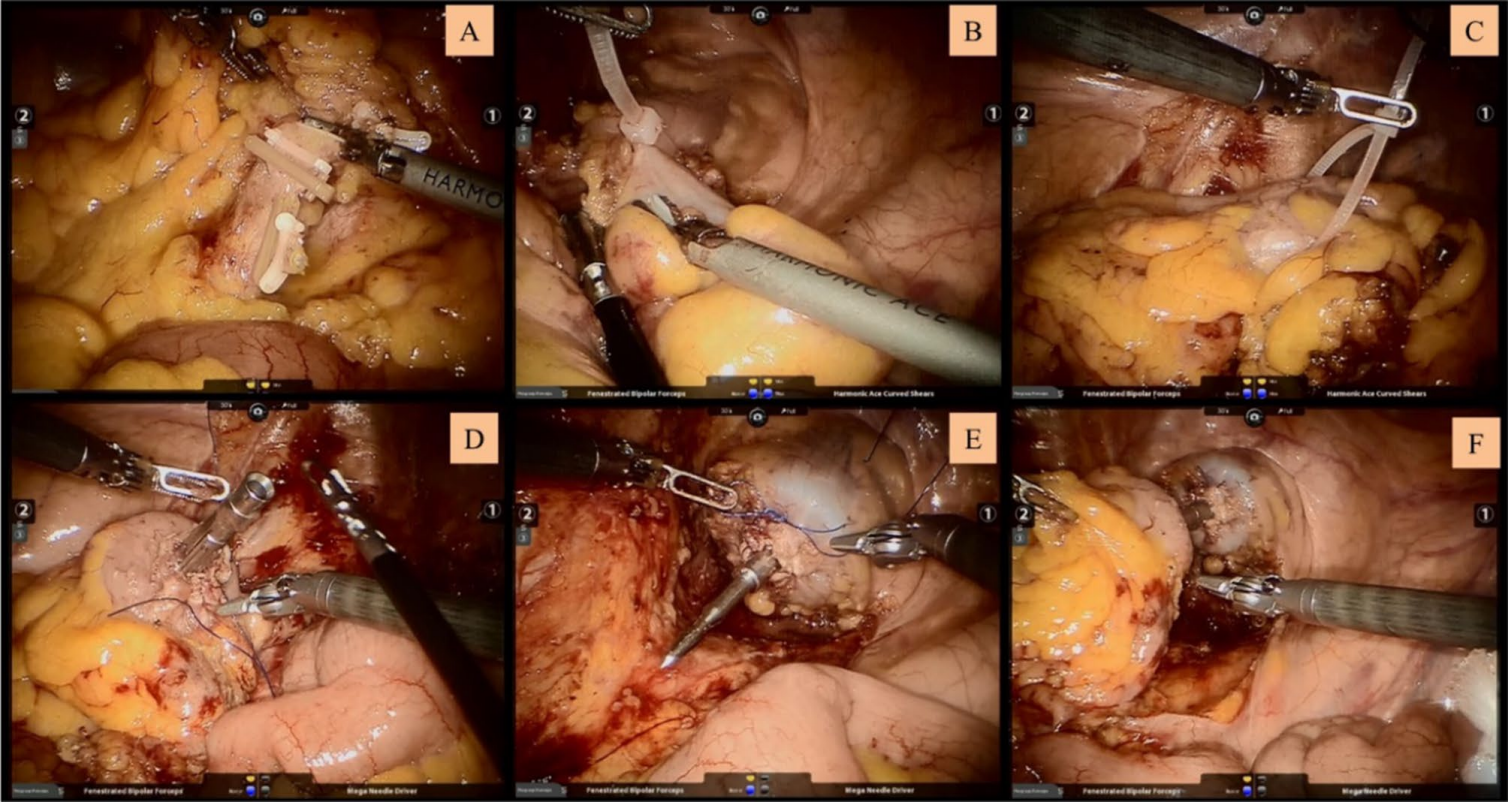
Surgical procedure. (**A**) The inferior mesenteric artery and vein were isolated and clipped by absorbable vascular clamps; (**B**) Rectum was ligated with self-locking nylon bandage; (**C**) Sigmoid colon was ligated with self-locking nylon bandage; (**D**) Suture the stump of sigmoid colon and put the orvil into the sigmoid colon; (**E**) Suture the stump of rectum; (**F**) Complete the anastomosis.

After successful general anesthesia, the patient assumed the Trendelenburg position. Five trocars were used (Fig. 1): one 12 mm trocar located at 3 cm above the umbilicus for robotic camera and another 12 mm trocar located at the right midclavicular line 1 cm above the umbilicus for assistant; three 8 mm trocar for robotic arms (R1 located at the right anterior superior iliac spine, R2 located at the left midclavicular line 1 cm above the umbilicus, and R3 located at the left anterior superior iliac spine). First, an ultrasonic scalpel via R1 was used to separate the internal and external peritoneum of sigmoid colon, and the left ureter was properly protected. The inferior mesenteric artery and vein were isolated and clipped by absorbable vascular clamps (Fig. 1A). The left colic artery was preserved. The rectum was completely isolated until 2–5 cm to the lower edge of the tumor. Then, the colorectum was ligated with self-locking nylon bandage (Fig. 1B,C). After the rectum was cut off by an ultrasonic scalpel, the assistant inserted an endoscope-sterile sleeve for protection of the specimen into the pelvic cavity through the anus, and the resected specimen was pulled out through the anus. The assistant sent an orvil through the anus, and the operator sutured the stump of the sigmoid colon and placed the orvil into the sigmoid colon (Fig. 1D). After the operator sutured the stump of the rectum (Fig. 1E), the assistant placed the curved intraluminal stapler through the anus to complete the anastomosis (Fig. 1F). Then, the assistant injected air into the rectum through the anus. If air leakage occurred from the anastomosis or serous membrane eversion, suture can be performed. Perioperative management followed the international guidelines^8^.

### Statistical analysis

Data processing and statistical analysis were performed using SPSS (version 22.0, SPSS Inc., Chicago, IL, USA). Measurement data were expressed as the mean ± standard deviation, and an independent sample t-test or variance analysis was used to compare the mean values. Qualitative data were expressed as frequency and percentage, and Pearson’s χ^2^ test or Fisher’s exact probability method was used. P < 0.05 was considered statistically significant (two-tailed test).

### Institutional review board statement

This study was reviewed and approved by the Ethics Committee of the Second Xiangya Hospital, Central South University.

### Informed consent statement

Informed consent was obtained from all patients for using their clinical data in the study.

## Results

### Patient characteristics

This study included 180 patients. In terms of gender, men accounted for 60% (108/180) and women for 40% (72/180). The mean age was (57.3 ± 13.1) years old, and the mean body mass index (BMI) was (23.6 ± 3.3) kg/m^2^. The most common chief complaint was hematochezia in 81.1% of patients (146/180). 165 patients were tested for carcinoembryonic antigen (CEA) and carbohydrate antigen (CA) 242, and 133 patients were tested for CA19-9 before operation. The distance from the lower edge of the tumor to the anus was based on the colonoscopy report, with an average of (8.64 ± 3.64) cm; those measuring less than 5, 5–10, and greater than or equal to 10 cm accounted for 7.2% (13/180), 54.4% (98/180), and 38.3% (69/180), respectively. The proportion of patients with nRCT was 15% (27/180). Twenty-six patients had abdominal or pelvic surgery history (14.4%). American Society of Anesthesiologists (ASA) classification levels 1, 2, 3, and 4 reached 3.3% (6/180), 50.5% (91/180), 44.4% (80/180), and 1.7% (3/180) of patients, respectively (Table 1).

**Table 1:** Patient characteristics.

	Cases (n)	Ratio (%)
**Gender**		
Male	108	60
Female	72	40
**Age**		
20–39	16	8.9
40–59	79	43.9
60–79	75	41.7
≥ 80	10	5.6
**Chief complaint**		
Hematochezia	146	81.1
increased times in defecation	25	13.9
Abdominal discomfortable	2	1.1
Anal distention	2	1.1
Routine examination	5	2.8
**Systemic disease**		
Hypertension	41	22.8
Diabetes	16	8.9
Cardiovascular diseases	10	5.6
Respiratory diseases	6	3.3
Cerebrovascular disease	7	3.9
Cirrhosis	5	2.8
Other	9	5
**ASA score**		
1	6	3.3
2	91	50.5
3	80	44.4
4	3	1.7
**History of abdominal surgery**		
Yes	26	14.4
No	154	85.6
**nCRT**		
Yes	27	15
No	153	85
**Distance from the lower edge of the tumor to the anus**		
< 5 cm	13	7.2
5-10 cm	98	54.4
≥ 10 cm	69	38.3
**CEA(ng/mL)**		
Normal	136	82.4
Higher	29	17.6
**CA19-9(ng/mL)**		
Normal	124	93.2
Higher	9	6.8
**CA242(ng/mL)**		
Normal	157	95.2
Higher	8	4.8

### Postoperative pathological information

In this study, the most common histological types were tubular adenocarcinoma(170/180, 94.4%), and the most common degree of differentiation of tubular adenocarcinoma was moderately differentiated adenocarcinoma, accounting for 80.6% (137/170). The average CDmax of specimen was (3.5 ± 1.6) cm, and the highest CDmax value was 12 cm. The number of lymph nodes harvested in all cases ≥ 12 accounted for 73.9% (133/180), and the average number of lymph nodes harvested for each case was (14.8 ± 5). Based on the depth of tumor invasion, Tis stage was 1.2% (2/173), T1 stage was 8.1% (14/173), T2 stage was 26.6% (46/173), T3 stage was 37.6% (65/173), and T4 stage was 26.6% (46/173). Lymph node metastasis accounted for 33.5% (58/173), and simultaneous liver metastasis was observed in one case. No positive resection margin was observed (Table 2).

**Table 2:** Pathological information.

	Cases (n)	Ratio (%)
**Histology type**		
Tubular adenocarcinoma	170	94.4
Mucinous adenocarcinoma	3	1.7
Adenoma	5	2.8
Endometriosis	1	0.6
Neuroendocrine tumor	1	0.6
**Differentiation**		
Highly	3	1.8
Moderately	137	80.6
Poorly	30	17.6
**CDmax**		
< 3 cm	59	32.8
3-5 cm	84	46.7
≥ 5 cm	37	20.6
**T staging**		
Tis	2	1.2
T1	14	8.1
T2	46	26.6
T3	65	37.6
T4	46	26.6
**Lymph node harvested**		
< 12	47	26.1
≥ 12	133	73.9
**Lymph node metastasis**		
Yes	58	33.5
No	115	66.5
**Liver metastasis of adenocarcinoma**		
Yes	1	0.6
No	172	99.4
**Resection margins**		
Positive	0	0
Negative	180	100

### Perioperative outcomes

All 180 cases completed robotic NOSES successfully, and no conversion to laparotomy was observed. The average medical cost was 110,046 ± 35,003 CNY. The mean operative time was (187.5 ± 78.3) min, and the mean intraoperative blood loss was (47.4 ± 34) mL. Most patients underwent robotic TME for the rectum or complete mesocolic excision (CME) for the sigmoid (171/180). All specimens were removed through the anus. Cases for protective ileostomy accounted for 6.7% (12/180). The average postoperative fasting time was (4.2 ± 3.9) days, and the average postoperative hospital stay was (11.3 ± 7.5) days. The incidence of postoperative complications was 11.7% (21/180). The incidence of anastomotic leakage was 4.4% (8/180), and the other complications included abdominal infection, lung infection, anastomotic bleeding, and abdominal hemorrhage. The reoperation rate was 2.2% (4/180), which was achieved with ileostomy in all cases after anastomotic leakage. The other 17 patients with complications were managed with anti-infective therapy, ultrasound-guided percutaneous drainage, colonoscopy, and endoscopic treatment. No postoperative anal dysfunction nor 90-day death was observed (Table 3).

**Table 3:** Perioperative outcomes.

	Cases (n)	Ratio (%)
**Surgical procedure**		
TME or CME	171	95
TME + Subtotal colectomy	1	0.6
TME + Resection of liver metastasis	1	0.6
TME + Resection of partial transverse colon	1	0.6
TME + Ovariectomy	2	1.1
TME + Hysterectomy	2	1.1
TME + Left partial nephrectomy	1	0.6
TME + Ureteral repair	1	0.6
**Protective ileostomy**		
Yes	12	6.7
No	168	93.3
**Clavien-Dindo classification**		
I	1	0.6
II	10	5.6
IIIa	6	3.3
IIIb	3	1.7
IVb	1	0.6
**Anastomotic leakage**		
Yes	8	4.4
No	172	95.6
**Reoperation**		
Yes	4	2.2
No	176	97.8
**Postoperative anal function**		
Abnormal	0	0
Normal	180	100
**90-day mortality**		
Yes	0	0
No	180	100

### Influence on robotic NOSES by the distance between tumor and anus or nRCT

The safety and feasibility were compared between three groups based on the distance from the lower edge of the tumor to the anus. Table 4 shows the results. Close distance from the lower edge of the tumor to the anus means long operation time, high intraoperative blood loss, and high incidence of anastomotic leakage. The difference was statistically significant (P < 0.05). nRCT had no effect on the safety and feasibility for robotic NOSES. The results are shown in Table 5.

**Table 4:** Influence on robotic NOSES by the distance between the lower edge of the tumor to the dentate line.

	< 5 cm	5–10 cm	≥ 10 cm	P
**Safety**				
Operative time (min)	263.9 ± 178.6	184 ± 61.9	178 ± 61.7	0.001
Blood loss (mL)	54.6 ± 48.4	45.2 ± 29.8	49.3 ± 36.8	0.546
Conversion to laparotomy (%)	0	0	0	N/A
Postoperative hospital stay (days)	16.5 ± 12.4	12 ± 8.3	9.4 ± 3.3	0.002
Anastomotic leakage (n, %)	1/13,7.7%	7/98,7.1%	0/69,0%	0.048
Reoperation (n, %)	0,0%	4/98,4.1%	0,0%	0.266
90-day mortality (%)	0	0	0	N/A
**Feasibility**				
Lymph node harvested	13 ± 3.1	14.5 ± 5.3	15.5 ± 4.7	0.192
Positive resection margins (%)	0	0	0	N/A

**Table 5:** Influence on robotic NOSES by nRCT.

	With nRCT	No nRCT	P
**Safety**			
Operative time (min)	184.8 ± 76.1	187.9 ± 78.9	0.846
Blood loss (ml)	57.4 ± 39	45.7 ± 32.9	0.098
Conversion to laparotomy (%)	0	0	N/A
Postoperative hospital stay (days)	11.7 ± 5.8	11.3 ± 7.7	0.774
Anastomotic leakage (n, %)	6/158,3.8%	2/27,7.4%	0.076
Reoperation (n, %)	0,0%	4/153,2.6%	> 0.999
90-day mortality (%)	0	0	N/A
**Feasibility**			
Lymph node harvested	14 ± 5.6	14.9 ± 4.9	0.423
Positive resection margins (%)	0	0	N/A

## Discussion

In 2007, French doctor Marescaux completed the first truly scar free operation in the world, and transvaginal cholecystectomy, which minimally invasive surgery requirements, has entered a new era^3^. NOSES is a kind of operation that can realize the concept of “no scar” surgery to a certain limit. It is especially suitable for colorectal surgery. Incisions in the oral cavity, rectum, vagina, and other natural orifices for appendectomy, cholecystectomy, and nephrotomy are often necessary to remove specimens from the natural lumen. The rectum has to be disconnected during colorectal surgery. The rectum and anus could be natural orifices for specimen extraction and could be used to avoid performing any artificial incision, rendering them with evident natural advantage. Based on the orifice for specimen extraction, NOSES could be divided into transanal and transvaginal NOSES. However, all cases presented in these reports were transanal NOSES. We excluded the specimen removal from the vagina for the following reasons. First, transvaginal NOSES could only be applicable for female patients. Second, incision on the vaginal wall may increase the risk of postoperative complications and sexual dysfunction. Third, transvaginal NOSES is also limited by ethics. In June 2017, China NOSES Alliance was established and released *Expert consensus of natural orifice specimen extraction surgery in colorectal neoplasm (2017 edition)*^9^ to promote the application of NOSES. The International Alliance of NOSES also issued *International consensus on natural orifice specimen extraction surgery (NOSES) for colorectal cancer* in 2019^7^.

Many reports are available on laparoscopic NOSES for colorectal cancer^4,10^; compared with conventional laparoscopic surgery, laparoscopic NOSES is a safe procedure and can achieve similar oncological outcomes. Wolthuis et al. conducted a clinical trial comparing the short-term effects of conventional laparoscopic surgery and laparoscopic NOSES; their results showed that the postoperative pain of patients in the NOSES group was alleviated significantly^11^. According to the report of an international multicenter study including 412 cases of conventional laparoscopic radical resection of rectal cancer and 356 cases of laparoscopic NOSES, no difference was observed in the operation time, the number of lymph nodes harvested, incidence of anastomotic leakage, and length of postoperative hospital stay. Incision infection and incisional hernia were also observed in the conventional laparoscopic radical resection group^10^. A retrospective study of 718 cases of colorectal cancer in 79 hospitals in China showed the following results: incidence of anastomotic leakage of 3.5%, reoperation rate of 3.6%, and average intraoperative blood loss less than 100 mL; these findings fully demonstrated the safety of laparoscopic NOSES for rectal cancer^12^. Two meta-analysis involving 1435 and 837 patients also showed that compared with conventional laparoscopic surgery, NOSES may be a safe procedure and can significantly reduce the duration of hospital stay, accelerate the postoperative recovery with good cosmetic results, result in less postoperative pain and limited complications, and achieve similar oncological outcomes^13,14^.

Numerous studies have reported robot-assisted radical resection of colorectal cancer^10,15–19^. However, in most reports, the specimens were extracted through a small abdominal incision; meanwhile, the reports on robotic NOSES are rare. Three retrospective analysis reports exist, and the rest are case reports^20–23^. Compared with the previous reports on laparoscopic NOSES, robotic NOSES in our hospital revealed advantages in terms of safety and feasibility^5,12^. In terms of the safety of robotic NOSES, our results showed that the average operation time was (187.5 ± 78.3) min, intraoperative blood loss was (47.4 ± 34) mL, no conversion to laparotomy occurred, postoperative hospital stay was (11.3 ± 7.5) days, the incidence of anastomotic leakage was 4.4%, the reoperation rate was 2.2%, and no 90-day death was observed. In terms of the feasibility, the average number of lymph nodes harvested was (14.8 ± 5), and no case with positive margin was observed (Table 6).

**Table 6:** Comparison between robotic NOSES and laparoscopic NOSES.

	Robotic NOSES	Wang^5^	Xu^12^
**Safety**			
Operative time (min)	187.5 ± 78.3	198.9 ± 55.2	210.5 ± 39.4
Blood loss (mL)	47.4 ± 34	73.7 ± 54.2	61.8 ± 23.1
Conversion to laparotomy (%)	0	0	0
Postoperative hospital stay (d)	11.3 ± 7.5	12.3 ± 4.1	12.1 ± 4.0
Anastomotic leakage (n, %)	8,4.4%	9,4.43%	25,3.5%
Reoperation (n, %)	4,2.2%	2,1%	23,3.2%
90-day mortality (%)	0	0	0
**Feasibility**			
Lymph node harvested	14.8 ± 5	12.9 ± 5.1	13.4 ± 3.5
Positive resection margins (%)	0	0	0

Either laparoscopic or robotic NOSES could be performed in our department^24,25^. As per our experience, especially in ultra-low rectal cancer, robotic NOSES has advantage compared with laparoscopic NOSES. For ultra-low rectal tumor, if the rectum is cut off directly by an ultrasonic scalpel during laparoscopic surgery, suturing of the pouch is difficult. However, if the rectum is to be amputated with an endoscopic linear cutter, the procedure may not be completed due to the narrow pelvic cavity. However, the problems mentioned above could be resolved by robotic NOSES. The ultra-low rectum can be sutured directly after the rectum is amputated by an ultrasonic scalpel. If the anastomosis is unsatisfactory, we can use the da Vinci Robot System to sew and strengthen the anastomosis directly to reduce the incidence of anastomotic leakage. However, it is important to realize that a close distance from the lower edge of the tumor to the anus with long operation time and postoperative hospital stay indicates high incidence of anastomotic leakage. In addition, either received nRCT has no effect on perioperative safety.

This study showed that T4 stage cases accounted for 26.6%, and the proportion of patients whose maximum diameter of tumor is greater than or equal to 5 cm was 20.6%, which is inconsistent with the recommendations of the *International consensus on natural orifice specimen extraction surgery (NOSES) for colorectal cancer*^7^. Based on the recommendations, cases of T2 and T3 stage tumors should be appropriate for NOSES, whereas T4 stage cases are not recommended given the difficulty of guaranteeing a negative CRM. However, we believe that for experienced teams in colorectal minimally invasive surgery, robotic NOSES for T4 stage cases is safe while strictly observing the principle of tumor-free technique. In terms of the CDmax of specimen, the consensus suggested that if the specimen is to be removed through the anus, then the CDmax should be less than 3 cm. However, our data show that the proportion of CDmax of specimen exceeding 3 cm accounted for 67.3%, that exceeding 5 cm accounted for 20.6%, and the largest reached 12 cm. We believe that if CDmax is limited to specimens not exceeding 3 cm, more than a half of patients will lose the opportunity for NOSES. Based on our experience, indications for NOSES could be appropriately relaxed. On the premise of full anal dilation, most specimens with CDmax of less than 5 cm can be removed through the anus without specimen damage. This procedure will not lead to laceration of the rectum and anus. In addition, the specific situation should be analyzed in detail. When the specimens are extracted from the anus during NOSES, one end of the rectum is clamped by surgical forceps, and the specimens are extracted from the anus along the longitudinal axis of the rectum. Therefore, if the CDmax is along the longitudinal axis of the rectum, specimens can still be easily extracted from the anus despite a CDmax exceeding 5 cm. On the contrary, a CDmax vertical to the longitudinal axis of the rectum will increase the difficulty of pulling out the specimen from the anus. In this study, the largest CDmax of specimen extracted from the anus was 12 cm. However, this length was measured along the longitudinal axis of the rectum, and its short axis was less than 2 cm. Thus, the specimen was a long strip that can be naturally removed through the anus. Whether the specimen can be extracted from the anus is also affected by other factors. For example, if the distance between the lower edge of the tumor and the anus is relatively close, dilation of the rectum, which needs to be protected during specimen extraction, is unnecessary. Thus, the CDmax of specimens could be large. Otherwise, if the distance is relatively long, the free rectum in the pelvic cavity must be dilated during specimen extraction, which will lead to laceration of the rectum and anus if the CDmax of specimen is notably large. As a promising minimally invasive technique, we appeal to all surgical colleagues working on robotic NOSES to promote the development of robotic NOSES in the world for the benefit of colorectal patients.

Our analysis has several limitations. First, progression-free survival and overall survival of all selected cases need to be verified through a long follow-up. Second, this research is a retrospective study. Thus, the integrity and homogeneity of research data cannot be guaranteed.

## Data Availability

No additional data are available.

## References

[1] Marks JH, Huang R, McKeever D (2016). Outcomes in 132 patients following laparoscopic total mesorectal excision (TME) for rectal cancer with greater than 5-year follow-up. Surg. Endosc..

[2] Cho MS, Kim CW, Baek SJ (2015). Minimally invasive versus open total mesorectal excision for rectal cancer: Long-term results from a case-matched study of 633 patients. Surgery.

[3] Zorron R, Filgueiras M, Maggioni LC (2007). NOTES. Transvaginal cholecystectomy: Report of the first case. Surg. Innov..

[4] Park JS, Kang H, Park SY (2018). Long-term outcomes after Natural Orifice Specimen Extraction versus conventional laparoscopy-assisted surgery for rectal cancer: A matched case-control study. Ann. Surg. Treat. Res..

[5] Yuliuming W, Qian Z, Lei Y (2019). Retrospective study of 203 cases of colorectal neoplasms treated by natural orifice specimen extraction surgery. Chin. J. Colorectal. Dis..

[6] Yao H, Li T, Chen W (2020). Safety and feasibility of robotic natural orifice specimen extraction surgery in colorectal neoplasms during the initial learning curve. Front. Oncol..

[7] Guan X, Liu Z, Longo A (2019). International consensus on natural orifice specimen extraction surgery (NOSES) for colorectal cancer. Gastroenterol. Rep. (Oxf).

[8] Gustafsson UO, Scott MJ, Hubner M (2019). Guidelines for perioperative care in elective colorectal surgery: Enhanced recovery after surgery (ERAS((R))) Society Recommendations: 2018. World J. Surg..

[9] Alliance CN (2017). Expert consensus of natural orifice specimen extraction surgery in colorectal neoplasm (2017 edition). Chin. J. Colorectal Dis..

[10] Liu Z, Efetov S, Guan X (2019). A multicenter study evaluating natural orifice specimen extraction surgery for rectal cancer. J. Surg. Res..

[11] Wolthuis AM, Fieuws S, Van Den Bosch A (2015). Randomized clinical trial of laparoscopic colectomy with or without natural-orifice specimen extraction. Br. J. Surg..

[12] Xu G, Qingchao T, Guiyu W (2017). Retrospective study of 718 colorectal neoplasms treated by natural orifice specimen extraction surgery in 79 hospitals. Chin. J. Colorectal Dis..

[13] Liu RJ, Zhang CD, Fan YC (2019). Safety and oncological outcomes of laparoscopic NOSE surgery compared with conventional laparoscopic surgery for colorectal diseases: A meta-analysis. Front. Oncol..

[14] Ma B, Huang XZ, Gao P (2015). Laparoscopic resection with natural orifice specimen extraction versus conventional laparoscopy for colorectal disease: A meta-analysis. Int. J. Colorectal Dis..

[15] Mian W, Qian-jin L, Jian-yong Z (2015). Comparison of short-term outcomes between robotic-assisted and laparoscopic proctectomy for rectal cancer: A case-control study. Chin. J. Colorectal. Dis..

[16] Cao C-L, Li T-Y, Liu D-N (2016). Comparison of short-term outcomes between roboticassisted and laparoscopic surgery for rectal cancer. World Chin. J. Digestol..

[17] Zhen ZOU, Bo T, Dongning L (2018). Short-term outcomes of robotic versus laparoscopic radical resection for middle and low rectal cancer: A single-center randomized, controlled study. Chin. J. Gen. Surg..

[18] Liu WH, Yan PJ, Hu DP (2019). Short-term outcomes of robotic versus laparoscopic total mesorectal excision for rectal cancer: A cohort study. Am. Surg..

[19] Megevand JL, Lillo E, Amboldi M (2019). TME for rectal cancer: Consecutive 70 patients treated with laparoscopic and robotic technique-cumulative experience in a single centre. Updates Surg.

[20] Xuefeng Z, Chi L, Cheng Z (2013). Totally robotic surgery for rectal cancer with transanal specimen extraction. Chin. J. Pract. Surg..

[21] Hu R, Jiang L, Kun Z (2014). Trans-natural orifice transuminal specimen extraction in robotic rectal cancer surgery: An analysis of 21 patients. Chin. J. Pract. Surg..

[22] Efetov SK, Tulina IA, Kim VD (2019). Natural orifice specimen extraction (NOSE) surgery with rectal eversion and total extra-abdominal resection. Tech. Coloproctol..

[23] Minjares-Granillo RO, Dimas BA, LeFave JJ (2019). Robotic left-sided colorectal resection with natural orifice IntraCorporeal anastomosis with extraction of specimen: The NICE procedure. A pilot study of consecutive cases. Am. J. Surg..

[24] Zhou J, Xiong L, Miao X (2020). Outcome of robot-assisted pancreaticoduodenectomy during initial learning curve versus laparotomy. Sci. Rep..

[25] Zhou JJ (2020). Analysis of robotic natural orifice specimen extraction surgery on 162 cases with rectal neoplasms. Zhonghua weichang waike za zhi [Chin J Gastrointest Surg].

